# Efficacy of part-time patching in preventing recurrence after bilateral lateral rectus recession in children with intermittent exotropia

**DOI:** 10.1186/s12886-023-03259-8

**Published:** 2023-12-14

**Authors:** Jae Yong Han, Jinu Han, Sueng-Han Han

**Affiliations:** Department of Ophthalmology, Institute of Vision Research, Severance Hospital, Yonsei University College of Medicine, 50-1, Yonseiro, Seodaemun-gu, Seoul, Republic of Korea

**Keywords:** Intermittent exotropia, Part-time patching, Recurrence, Reoperation

## Abstract

**Background:**

This study evaluate the efficacy of part-time patching in preventing recurrence after bilateral lateral rectus recession (BLR) in patients with intermittent exotropia (IXT).

**Methods:**

A total of 190 children aged 3–13 years who experienced recurrence after BLR for IXT and received part-time patching were retrospectively reviewed. The patching was prescribed for 2 h per day for more than 6 months. Patients who had a recurrence of 18 PD or more underwent reoperation. Changes in exodeviation and reoperation ratio after part-time patching were analyzed.

**Results:**

A total of 34 patients (17.9%) received reoperation after part-time patching, and the reoperation ratio after 2 years was 20.3% as per the Kaplan–Meier survival analysis. Patients with a recurrence of 7 to 10 PD showed a significantly better effect compared to those with a recurrence of more than 10 PD (*p* < 0.001), and the reoperation ratio was also lower in the survival analysis (*p* = 0.004). The factor associated with reoperation in patients with part-time patching was the duration between the operation and the initiation of part-time patching (hazard ratio [HR] = 1.006, *p* = 0.002).

**Conclusions:**

Part-time patching was effective in maintaining the efficacy of surgery and delaying the need of reoperation after BLR. This effect was better in patients with a recurrence of ≤ 10 PD.

**Supplementary Information:**

The online version contains supplementary material available at 10.1186/s12886-023-03259-8.

## Background

Intermittent exotropia (IXT) is one of the most common types of strabismus in children, affecting as much as 1% of the population [1], and is more prevalent in Asian countries [2, 3]. IXT is a disease characterized by intermittent exodeviation, exacerbated by fatigue, disease and daydreaming. There are surgical and non-surgical treatments for IXT, and part-time patching is one of the most representative non-surgical treatments [4–6]. Part-time patching is used as an anti-suppression therapy to stimulate motor fusion in the treatment of IXT [6–8]. Various investigations conducted on the therapeutic effect of part-time patching range from studying the mechanism to confirming appropriate patching times. In these reports, part-time patching has been shown to preserve binocularity, reduce the frequency and amount of exodeviation, improve control, and even reduce the size of the suppression scotoma [6]. Moreover, part-time patching has been reported to delay or even prevent surgery [6–11].

In previous studies, part-time patching has mostly been compared to surgery as a treatment for IXT. To the best of our knowledge, no study has reported part-time patching as a tool to prevent recurrence and reoperation after surgery for IXT. We expect that stimulation of motor fusion through anti-suppression would decrease the recurrence rate and inhibit its progression after surgery, as well as being effective prior to surgery. The study aimed to determine whether part-time patching after surgery can maintain the therapeutic effect of surgery and reduce reoperation due to recurrence.

## Methods

### Study populations

The study protocol was approved by the institutional review board of Severance Hospital, Yonsei University College of Medicine, Korea (4-2022-1640). Owing to the retrospective design of the study, the need for informed consent was waived by the Severance Hospital Institutional Review Board. This study was performed in accordance with the tenets of the Declaration of Helsinki.

The medical records of children aged 3–13 years who underwent part-time patching after bilateral lateral rectus recession (BLR) for IXT between March 2016 and June 2022 were retrospectively reviewed. Patients who experienced a recurrence of more than 7 prism diopters (PD) of exodeviation and were prescribed part-time patching for more than 6 months were included. Patients with recurrence of ≥ 18 PD exodeviation with poor control underwent reoperation. Patients with poor cooperation during alternate cover testing with prism at distance or near conditions were excluded. Patients with amblyopia, severe refractive errors (< -6.50 D or > + 5.00 D), severe ocular diseases in addition to IXT, developmental delay, and any other systemic or neurologic diseases associated with strabismus were also excluded.

### Study design

Patching was prescribed in the patients’ dominant eye for 2 h per day. If the patients showed alternating fixation, it was decided to cover the more fixating eye after interviewing the patients or guardians. Compliance with patching was assessed based on parental history at each visit.

All patients underwent cycloplegic refraction, alternative prism cover test, office control check, and tests for stereoacuity at the preoperative visit. Alternative cover test and office control check were performed for all participants at each follow-up visit. The angle of deviation was assessed by a single ophthalmologist (S.H.H), using the alternative prism cover test at both distance (6 m) and near (33 cm) conditions under full correction of refractive errors. Office-based control was also assessed by a single ophthalmologist (S.H.H) in the clinic. (1) If the fusion broke only after cover testing and resumed rapidly without blinking or refixation, the control was ‘good’. (2) If the patient resumed fusion after blinking or re-fixation after disruption with cover testing, the control was ‘fair’. (3) ‘Poor’ control was defined as strabismus presenting most of the time, easy to break down, and did not recover well after occlusion [12]. Titmus stereotest (Stereo Optical Co., Chicago, IL, USA) and Worth 4-dot tests were performed for stereoacuity. Due to poor cooperation, stereoacuity was collected only from 170 patients. The Titmus test was conducted at a distance of 40 cm from the subjects while wearing polarizing glasses. During the examination, the examiner ensured a constant distance between the subject’s eye and the stereogram. The test consists of three portions: fly, animal, and circle, and can estimate stereopsis from 40 to 3000 arcsec. If the largest disparity could not be passed, stereoacuity was suggested as ‘nil stereo’ and was scored as 6000 arcsec to perform statistical analysis. For statistical analysis, all values were transformed to the logarithm of arc seconds [13, 14]. The Worth 4-dots test was performed at near (33 cm) and a distance (6 m) with the subject wearing red-green glasses. The results of the Worth 4-dot test were as follows: (1) fusion, if the lights were four; (2) suppression, if the lights were two or three; and (3) diplopia, if the lights were five [15].

All the BLRs were performed by the single surgeon (S.H.H). Based on the maximal angle of deviation during the pre-operative follow-up, BLR was performed in all patients using a surgical formula based on the surgeon’s experience (Supplementary Table 1).

To assess the difference based on the angle of deviation, we classified the patients into two groups based on exodeviation before initiation the part-time patching: those with exodeviation of 7 to 10 PD were categorized as low recurrence and patients with exodeviation of more than 10 PD were categorized as high recurrence group. Moreover, the patients were classified into two groups based on the reoperation: the reoperated group and the non-reoperated group. Individual analyses were conducted for each pair of groups.

### Statistical analyses

Statistical analyses were performed using IBM SPSS for Windows (version 26.0; IBM Corp., Armonk, NY, USA). Statistical significance was set at *p* < 0.05. The paired t-test, independent t-test, chi-square test, Fisher’s exact test, and Mann–Whitney test were performed for statistical comparison. To determine the cumulative survival rate of reoperation after part-time patching, Kaplan–Meier curves were plotted. Univariate analysis was performed for each variable to determine the factors related to reoperation, and variables with *p*-value < 0.3 in univariate analysis, were included in the multivariate Cox proportional hazards model. Linear mixed models (LMM) were performed for the repeatedly measured angle of deviation to determine the difference between the low and high recurrence groups and the reoperated and non-reoperated groups. Due to the different numbers and random intervals of follow-up, the follow-up interval was set as a random effect. Variables with a significant *p*-value (*p* < 0.3) in the univariate LMM were used for multivariate LMM. The variables were as follows: age, preoperative exodeviation, Worth 4-dot test at a distance, operation amount, pre-patching exodeviation, pre-patching office control, and the interval between operation and patching. A generalized estimating equation (GEE) was used for the analysis of categorical variables.

## Results

The demographics and baseline characteristics of the subjects are shown in Table 1, including both pre-operative and pre-patching baseline characteristics. A total of 190 participants were enrolled in this study. Ninety patients (47.4%) were male and 100 patients (52.6%) were female. The mean age at operation was 5.89 ± 2.22 years. The preoperative angle of deviation was 29.36 ± 6.34 PD at near and 29.15 ± 6.32 PD at distance. In the Worth 4-dot test, four (2.3%) had diplopia, 66 (38.9%) had suppression, and 100 (58.8%) had fusion at near conditions. In the case of distance, nine (5.3%) had diplopia, 103 (60.6%) had suppression, and 58 (34.1%) had fusion. The mean score of the titmus test was 2.33 ± 0.64 log arcsec. There were 20 (10.5%) patients with poor control and 170 (89.5%) with fair control at preoperative evaluation. However, there was no patient with good control before the operation. The mean operation amount of BLR was 6.68 ± 0.98 mm. The mean angle of deviation at baseline evaluation before part-time patching was 10.74 ± 2.52 PD at the near condition and 10.66 ± 2.62 PD at distance. In contrast to the preoperative baseline characteristics, none of the patients exhibited poor control during the pre-patching evaluation. Besides, 182 (95.8%) patients had fair control and 8 (4.2%) had good control. The mean interval from the operation to recurrence was 58.72 ± 60.51 weeks, and for part-time patching, it was 64.94 ± 63.68 weeks. The mean duration of part-time patching was 68.08 ± 37.04 weeks, and the mean duration of follow-up was 78.33 ± 42.27 weeks. Despite part-time patching, 34 patients (17.9%) underwent reoperation (Table 1).

**Table 1:** Demographics and Baseline characteristics of participants

Characteristics	Values
Male:Female, n	90:100
Mean age at operation, yrs, mean ± SD (range)	5.89 ± 2.22 (3.0 to 14.0)
Preoperative baseline characteristics	
Exodeviation, PD, mean ± SD (range)	
Near	29.36 ± 6.34 (16 to 50)
Distance	29.15 ± 6.32 (18 to 50)
Worth 4-dot test, n (%) ^a^	
Near	
Diplopia	4 (2.3)
Suppression	66 (38.9)
Fusion	100 (58.8)
Distance	
Diplopia	9 (5.3)
Suppression	103 (60.6)
Fusion	58 (34.1)
Titmus test, log arcsec, mean ± SD (arcsecond, range) ^a^	2.33 ± 0.64 (40 to 6000)
Office control ,n (%)	
Poor	20 (10.5)
Fair	170 (89.5)
Good	0 (0)
Operation amount, mm, mean ± SD (range)	6.68 ± 0.98 (5.0 to 10.0)
Pre-patching baseline characteristics	
Best corrected visual acuity, LogMAR, mean ± SD (range)	
Right	0.05 ± 0.07 (0 to 0.22)
Left	0.05 ± 0.07 (0 to 0.22)
Exodeviation, PD, mean ± SD (range)	
Near	10.74 ± 2.52 (8 to 18)
Distance	10.66 ± 2.62 (8 to 18)
Office control, n (%)	
Poor	0 (0)
Fair	182 (95.8)
Good	8 (4.2)
The interval between operation and recurrence, weeks, mean ± SD (range)	58.72 ± 60.51 (4.7 to 253.0)
The interval between operation and patching, weeks, mean ± SD (range)	64.94 ± 63.68 (4.7 to 253.0)
Duration of part-time patching, weeks, mean ± SD (range)	68.08 ± 37.04 (24.0 to 201.7)
Duration of follow-up, weeks, mean ± SD (range)	78.33 ± 42.27 (24.5 to 250.6)
Reoperation, n (%)	34 (17.9)

yrs, years; SD, standard deviation; PD, prism diopters; n, numbers; LogMAR, Logarithmic Minimum angle of resolution; ^a^ the total number of subjects was 170 due to missing data.

The patients were divided into two groups according to the degree of recurrence. Between the two groups, only the reoperation ratio showed a significant difference (12.3% vs. 26.3%, *p* = 0.020) (Table 2). The changes in exodeviation over time were compared between the two groups by LMM and showed a significant difference. The high recurrence group showed a significantly greater increase in exodeviation at both near and at distance (*p* = 0.001 and *p* < 0.001, respectively). However, office control did not show a significant difference between the two groups in GEE.

**Table 2:** Comparison of Baseline characteristics in Low and High recurrence groups

Characteristics	Low recurrence (n = 114)	High recurrence (n = 76)	*p*-value
Male:Female, n	58:56	32:44	0.299
Mean age at operation, yrs, mean ± SD (range)	5.81 ± 2.11 (3.0 to 14.0)	6.01 ± 2.37 (3.1 to 13.2)	0.535
Preoperative baseline characteristics			
Exodeviation, PD, mean ± SD (range)			
Near	28.72 ± 6.42 (16 to 50)	30.33 ± 6.39 (20 to 45)	0.091
Distance	28.58 ± 6.07 (18 to 50)	30.00 ± 6.63 (20 to 45)	0.129
Worth 4-dot test, n (%) ^b^			
Near			0.199
Diplopia	1 (1.0)	3 (4.5)	
Suppression	38 (36.5)	28 (42.4)	
Fusion	65 (62.5)	35 (53.1)	
Distance			0.965
Diplopia	5 (4.8)	4 (6.1)	
Suppression	63 (60.6)	40 (60.6)	
Fusion	36 (34.6)	22 (33.3)	
Titmus test, log arcsec, mean ± SD (arcsecond, range) ^b^	2.29 ± 0.65 (40 to 6000)	2.39 ± 0.62 (40 to 6000)	0.308
Office control, n (%)			0.810
Poor	13 (11.4)	7 (9.2)	
Fair	101 (88.6)	69 (90.8)	
Good	0	0	
Operation amount, mm, mean ± SD (range)	6.61 ± 0.98 (5.0 to 10.0)	6.79 ± 0.98 (5.0 to 9.0)	0.217
Pre-patching baseline characteristics			
Best corrected visual acuity, LogMAR, mean ± SD (range)			
Right	0.04 ± 0.06 (0 to 0.22)	0.05 ± 0.08 (0 to 0.30)	0.359
Left	0.05 ± 0.06 (0 to 0.22)	0.05 ± 0.08 (0 to 0.30)	0.605
Office control, n (%)			0.480
Poor	0	0	
Fair	108 (94.7)	74 (97.4)	
Good	6 (5.3)	2 (2.6)	
The interval between operation and recurrence, weeks, mean ± SD (range)	53.16 ± 57.95 (4.7 to 253.0)	67.06 ± 63.64 (4.7 to 230.4)	0.121
The interval between operation and patching, weeks, mean ± SD (range)	57.66 ± 60.65 (4.7 to 253.0)	75.86 ± 66.89 (4.7 to 230.4)	0.053
Duration of part-time patching, weeks, mean ± SD (range)	68.96 ± 38.34 (24.0 to 201.7)	66.77 ± 35.22 (24.7 to 179.0)	0.691
Duration of follow-up, weeks, mean ± SD (range)	80.64 ± 45.70 (24.5 to 250.6)	74.86 ± 36.55 (24.7 to 179.0)	0.358
Reoperation, n (%)	14 (12.3)	20 (26.3)	0.020 ^a^

yrs, years; SD, standard deviation; n, numbers; PD, prism diopters; LogMAR, Logarithmic Minimum angle of resolution; ^a^ indicates statistically significant values (*p* < 0.05) ^b^ for individual variables the number of subjects may not add up to the total number of each group due to missing data.

**Fig. 1:**
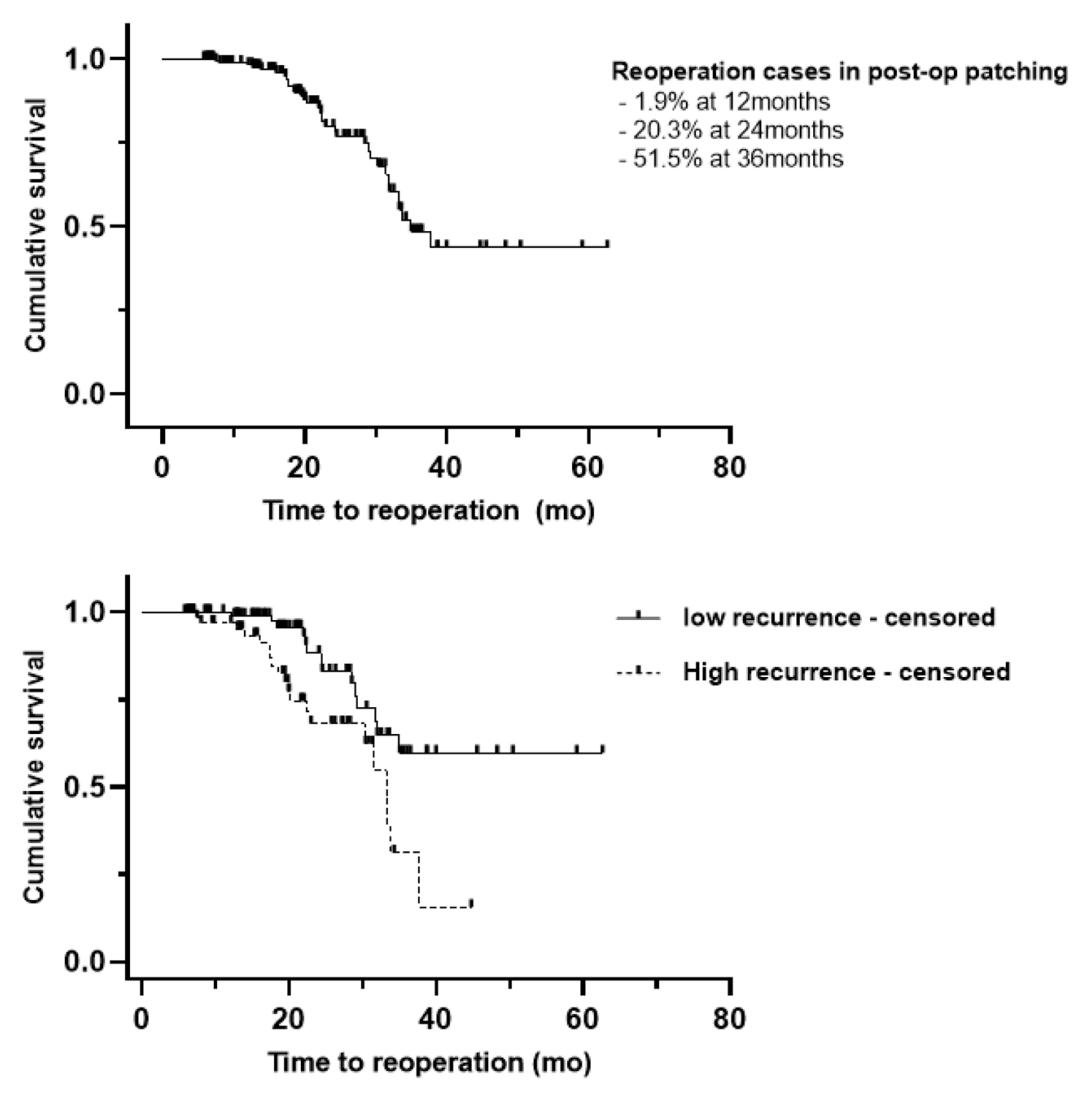
Kaplan–Meier survival analysis for reoperation after part-time patching. (Top) The total population of enrolled patients showed a 1.9% of reoperation ratio at 1 year, 20.3% at 2 years and 51.5% at 3 years. (Bottom) The low recurrence group showed a 1.1% of reoperation ratio at 1 year, 11.8% at 2 years, and 40.2% at 3 years. The high recurrence group showed relatively more reoperation: 3.1% of reoperation ratio at 1 year, 31.9% at 2 years, and 68.8% at 3 years. The *p*-value was 0.004 by log-rank test

Kaplan–Meier analysis was performed to reveal the risk of reoperation after part-time patching. The cumulative risk of reoperation among all patients was 1.9% at 12 months, 20.3% at 24 months, and 51.5% at 36 months. The low recurrence group showed a 1.1% reoperation ratio at 1 year, 11.8% at 2 years, and 40.2% at 3 years. The high recurrence group showed relatively more reoperation than the low recurrence group: 3.1% of reoperation ratio at 1 year, 31.9% at 2 years, and 68.8% at 3 years. This difference was statistically significant in the log-rank test (*p* = 0.004) (Fig. 1). The factors associated with reoperation in our study population were analyzed using the Cox proportional hazards model, and only the interval from operation to the initiation of part-time patching (hazard ratio [HR] = 1.006, *p* = 0.002) was significantly related in the multivariate analysis (Table 3).

**Table 3:** Univariate and multivariate cox proportional hazard models for factors associated with reoperation after ocular patching in patients with intermittent exotropia who underwent bilateral lateral rectus recession

	Univariate			Multivariate		
	HR	95% CI	*p*-value	HR	95% CI	*p*-value
Mean age at operation (yrs)	0.738	0.582–0.936	0.012			
Pre-patching exodeviation (near, PD)	1.241	1.073–1.436	0.004			
Pre-patching exodeviation (Distance, PD)	1.251	1.081–1.447	0.003			
The interval between operation and patching (wks)	1.006	1.000–1.011	0.037	1.006	1.001–1.011	0.023
Degree of recurrence ^a^	2.633	1.318–5.257	0.006			

HR, hazard ratio; CI, confidence interval; yrs, years; PD, prism diopters; wks, weeks; ^a^ Low and high recurrence group

To compare the characteristics associated with reoperation, we divided the study population into two groups: the reoperated group and the non-reoperated group. An additional analysis was conducted to examine these groups. There was a significant difference between the reoperated group and non-reoperated group in terms of mean age (*p* = 0.001) and the mean angle of deviation before patching, both at near (*p* = 0.012) and distance (*p* = 0.002). The interval between operation and recurrence (*p* = 0.038), interval between operation and the initiation of part-time patching (*p* = 0.034), duration of part-time patching (*p* = 0.003), and duration of total follow up (*p* = 0.038) were significantly different between the two groups (Table 4). The change in exodeviation over time was compared between the two groups by LMM and showed a significant difference; the reoperated group showed a significantly greater increase in exodeviation at both near and at distance (*p* < 0.001, respectively). Office control was poorer in the reoperated group than in the non-reoperated(*p* = 0.009) in GEE.

**Table 4:** Comparison of baseline characteristics in reoperated group and non-reoperated group

Characteristics	Reoperated (n = 34)	Non-reoperated (n = 156)	*p*-value
Male:Female, n	16:18	74:82	1.000
Mean age at operation, yrs, mean ± SD (range)	5.00 ± 1.39 (3.0 to 8.3)	6.08 ± 2.32 (3.3 to 14.0)	0.001 ^a^
Preoperative baseline characteristics			
Exodeviation, PD, mean ± SD (range)			
Near	30.15 ± 5.00 (25 to 40)	29.19 ± 6.71 (16 to 50)	0.435
Distance	30.00 ± 4.77 (25 to 40)	28.96 ± 6.61 (18 to 50)	0.290
Worth 4-dot test, n (%) ^b^			
Near			0.643
Diplopia	0 (0)	4 (2.8)	
Suppression	12 (41.4)	54 (38.3)	
Fusion	17 (58.6)	83 (58.9)	
Distance			0.273
Diplopia	0 (0)	9 (6.4)	
Suppression	17 (58.6)	86 (61.0)	
Fusion	12 (41.4)	46 (32.6)	
Titmus test, log arcsec, mean ± SD (arcsecond, range) ^b^	2.34 ± 0.65 (40 to 6000)	2.32 ± 0.64 (40 to 6000)	0.865
Office control, n (%)			0.776
Poor	3 (8.8)	17 (10.9)	
Fair	31 (91.2)	139 (89.1)	
Good	0 (0)	0 (0)	
Operation amount, mm, mean ± SD (range)	6.84 ± 0.68 (6.0 to 8.0)	6.65 ± 1.03 (5.0 to 10.0)	0.187
Pre-patching baseline characteristics			
Best corrected visual acuity, LogMAR, mean ± SD (range)			
Right	0.05 ± 0.07 (0 to 0.22)	0.05 ± 0.07 (0 to 0.3)	0.881
Left	0.05 ± 0.07 (0 to 0.22)	0.05 ± 0.07 (0 to 0.3)	0.745
Exodeviation, PD, mean ± SD (range)			
Near	11.91 ± 2.99 (25 to 40)	10.48 ± 2.35 (16 to 50)	0.012 ^a^
Distance	11.91 ± 2.99 (25 to 40)	10.39 ± 2.46 (18 to 50)	0.002 ^a^
Office control, n (%)			1.000
Poor	0 (0)	0 (0)	
Fair	33 (97.1)	149 (95.5)	
Good	1 (2.9)	7 (4.5)	
The interval between operation and recurrence, weeks, mean ± SD (range)	78.17 ± 55.53 (4.9 to 186.9)	54.48 ± 60.88 (4.7 to 253.0)	0.038 ^a^
The interval between operation and patching ,weeks, mean ± SD (range)	85.91 ± 59.36 (4.9 to 186.9)	60.37 ± 63.85 (4.7 to 253.0)	0.034 ^a^
Duration of part-time patching, weeks, mean ± SD (range)	85.07 ± 34.99 (26.0 to 151.0)	64.38 ± 36.54 (24.0 to 201.7)	0.003 ^a^
Duration of follow up, weeks, mean ± SD (range)	91.96 ± 31.94 (29.9 to 151.0)	75.36 ± 43.72 (24.5 to 250.6)	0.038 ^a^

yrs, years; SD, standard deviation; n, numbers; PD, prism diopters; LogMAR, Logarithmic Minimum angle of resolution; ^a^ indicates statistically significant values (*p* < 0.05); ^b^ for individual variables the number of subjects may not add up to the total number of each group due to missing data.

The comparison of best-corrected visual acuity between before patching and last follow-up has been added to Supplementary Table 2. Participants in this study showed a significant improvement in visual acuity after patching compared to their pre-patching condition. Furthermore, no other adverse effects related to patching were observed in the participants of this study.

## Discussion

The reoperation ratio after BLR has been previously reported in various studies. The reoperation ratio in our study was 17.9%. Choi and associates [16] reported that 8 (38.1%) of 21 patients who had a recurrence of more than 10 PD after BLR underwent reoperation. The Pediatric Eye Disease Investigator Group (PEDIG) [17] reported a randomized controlled trial comparing BLR with unilateral recess and resect for 3 years of follow-up. In this study, 7 (22.6%) of 31 patients who had a recurrence of more than 10 PD after BLR underwent reoperation. The reoperation ratio in our study was smaller than that in the aforementioned studies, which may indicate the effect of part-time patching. However, unlike the aforementioned studies, our study included patients who had a recurrence of less than 10 PD. In our study, 76 patients had recurrence of more than 10 PD, and the reoperation ratio was 26.3%, which is not much different from previous studies. Based on this result, the effect of part-time patching can be considered ambiguous. However, there are some reasons why the effect of part-time patching cannot be completely excluded. Firstly, there were more subjects in our study, and the criteria for reoperation were lower than those of the others. Simultaneously, the mean follow-up duration was longer than that of the others. It is well known that a longer follow-up duration is related to recurrence after BLR in IXT [18, 19]. These differences could have caused the high recurrence rate in our study.

Part-time patching showed a significantly greater effect on maintaining the angle of deviation in the low-recurrence group than in the other groups. In addition, the reoperation ratio was significantly lower in the low-recurrence group than in the high-recurrence group (12.3% vs. 26.3%). These differences between the two groups were more clearly observed in the survival analysis; the low-recurrence group showed a significantly higher survival rate, and this higher rate was maintained for more than 3 years (Fig. 1). Based on this result, it is expected that 2 h of part-time patching will have a better effect in patients with a recurrence of 10 PD or less.

Our study showed an improvement in best-corrected visual acuity after part-time patching (Supplementary Table 2). However, the individuals with amblyopia requiring occlusion therapy were excluded from this study. Furthermore, the improvement in visual acuity was not as prominent as typically observed in amblyopia occlusion therapy. Therefore, this is likely due to the ongoing development of visual acuity in relatively younger individuals among the study participants.

The factors associated with recurrence after surgery in IXT such as preoperative and postoperative degree of exodeviation, stereopsis, amblyopia, surgical procedures, and type of exotropia were previously reported [20]. Moreover, the age at the time of surgery was also reported to correlate with recurrence [21, 22]. In this study, there was a significant difference between the reoperation and the non-reoperation group in factors similar to those related to recurrence in previous studies (Table 4). The age and postoperative degree of exodeviation showed a significant correlation in univariate analysis, similar to previous reports. The degree of exodeviation at pre-patching and the starting point of patching also showed a significant correlation. However, in multivariate analysis, only the starting point of part-time patching showed a significant positive correlation (Table 3). However, the HR was only 1.006; therefore, the practical effects may be minimal.

Our study has some limitations due to the retrospective study design. Firstly, follow-up time and intervals differed among the participants. In particular, patients who underwent reoperation had more follow-up times and longer follow-up durations. In addition, these patients had a relatively longer patching duration than the others. This was due to the fact that the patients who underwent reoperation maintained part-time patching until just before the second operation. Stereoacuity and control also has problems due to the retrospective design. Unlike the angle of deviation, stereoacuity was only checked at the preoperative visit, and in some of the patients, stereoacuity was not checked due to poor co-operation of the patient. For the control group, the assessment method used in this study does not offer a quantitative representation. Subsequent research using quantitative methods may provide a more objective evaluation of changes in the control group. Thirdly, the patients who received only 2 h of part-time patching were enrolled in this study; therefore, there was no control group in this study: patients who did not receive patching or received patching other than 2 h. The lack of classification by type in intermittent exotropia (IXT) is also a limitation attributed to the retrospective nature of this study. It is necessary to investigate whether there are differences based on the type of IXT through additional research. Lastly, patch compliance in our study depended only on the patients’ statements. Therefore, there is a possibility of overestimation of patching time, and similarly, the effect of patching is likely to have been underestimated. To confirm the effect of part-time patching, a randomized prospective design would be needed for future research.

In conclusion, 2 h of part-time patching delayed reoperation and maintained the effectiveness of surgery in IXT patients with ≤ 10 PD recurrence. In patients with recurrence of > 10 PD, the effect of part-time patching was limited, although it still demonstrated some effect. Part-time patching would be a preceding option to consider before reoperation in recurrent IXT.

### Electronic supplementary material

Below is the link to the electronic supplementary material.

Supplementary Material 1

## Data Availability

The datasets used and/or analysed during the current study are available from the corresponding author on reasonable request.
