# Supplementary material for: Efficacy of part-time patching in preventing recurrence after bilateral lateral rectus recession in children with intermittent exotropia
Source: BMC Ophthalmol. 2023 Dec 14;23:510. doi: 10.1186/s12886-023-03259-8 (PMC10722733; doi:10.1186/s12886-023-03259-8)
Supplement: Supplementary file 1 — Supplementary Material 1 [file 12886_2023_3259_MOESM1_ESM.docx]

**Supplementary Table 1. Surgical table of bilateral lateral rectus recession in children**

| **Deviation (PD)** | **Surgical amount (mm)** |
| --- | --- |
| 15 | 4.5 |
| 20 | 5.5 |
| 25 | 6.5 |
| 30 | 7.0 |
| 35 | 7.5 |
| 40 | 8.0 |
| 45 | 9.0 |
| 50 | 10.0 |
| PD, prism diopter | |

| **Supplementary Table 2. Comparison of best corrected visual acuity before and after ocular patching** | | | |
| --- | --- | --- | --- |
|  | **Pre-patch BCVA** | **Post-patch BCVA** | **p-value** |
| Total (n=190, mean±SD) |  |  |  |
| Right | 0.05±0.07 | 0.00±0.02 | <0.001 |
| Left | 0.05±0.07 | 0.01±0.02 | <0.001 |
| Group by recurrence |  |  |  |
| Low recurrence (n=114, mean±SD) |  |  |  |
| Right | 0.04±0.06 | 0.00±0.02 | <0.001 |
| Left | 0.05±0.06 | 0.00±0.02 | <0.001 |
| High recurrence (n=76, mean±SD) |  |  |  |
| Right | 0.05±0.08 | 0.00±0.02 | <0.001 |
| Left | 0.05±0.08 | 0.01±0.03 | <0.001 |
| Group by reoperation |  |  |  |
| Reoperated (n=34, mean±SD) |  |  |  |
| Right | 0.05±0.07 | 0.00±0.01 | 0.001 |
| Left | 0.05±0.07 | 0.00±0.01 | <0.001 |
| Non-reoperated (n=156, mean±SD) |  |  |  |
| Right | 0.05±0.07 | 0.00±0.02 | <0.001 |
| Left | 0.05±0.07 | 0.01±0.03 | <0.001 |
| BCVA, best corrected visual acuity; n, numbers; SD, standard deviation; all values are expressed in Logarithmic Minimum angle of resolution. | | | |
|  | | | |
